# Dynamics of Genome Rearrangement in Bacterial Populations

**DOI:** 10.1371/journal.pgen.1000128

**Published:** 2008-07-18

**Authors:** Aaron E. Darling, István Miklós, Mark A. Ragan

**Affiliations:** 1 ARC Center of Excellence in Bioinformatics, The University of Queensland, St. Lucia, Queensland, Australia; 2 Institute for Molecular Bioscience, The University of Queensland, St. Lucia, Queensland, Australia; 3 Bioinformatics Group, Alfréd Rényi Institute of Mathematics, Hungarian Academy of Sciences, Budapest, Hungary; 4 eScience Regional Knowledge Centre, Eötvös Loránd University, Budapest, Hungary; 5 Data Mining and Search Research Group, Computer and Automation Institute, Hungarian Academy of Sciences, Budapest, Hungary; University of Toronto, Canada

## Abstract

Genome structure variation has profound impacts on phenotype in organisms ranging from microbes to humans, yet little is known about how natural selection acts on genome arrangement. Pathogenic bacteria such as *Yersinia pestis*, which causes bubonic and pneumonic plague, often exhibit a high degree of genomic rearrangement. The recent availability of several *Yersinia* genomes offers an unprecedented opportunity to study the evolution of genome structure and arrangement. We introduce a set of statistical methods to study patterns of rearrangement in circular chromosomes and apply them to the *Yersinia*. We constructed a multiple alignment of eight *Yersinia* genomes using Mauve software to identify 78 conserved segments that are internally free from genome rearrangement. Based on the alignment, we applied Bayesian statistical methods to infer the phylogenetic inversion history of *Yersinia*. The sampling of genome arrangement reconstructions contains seven parsimonious tree topologies, each having different histories of 79 inversions. Topologies with a greater number of inversions also exist, but were sampled less frequently. The inversion phylogenies agree with results suggested by SNP patterns. We then analyzed reconstructed inversion histories to identify patterns of rearrangement. We confirm an over-representation of “symmetric inversions”—inversions with endpoints that are equally distant from the origin of chromosomal replication. Ancestral genome arrangements demonstrate moderate preference for replichore balance in *Yersinia*. We found that all inversions are shorter than expected under a neutral model, whereas inversions acting within a single replichore are much shorter than expected. We also found evidence for a canonical configuration of the origin and terminus of replication. Finally, breakpoint reuse analysis reveals that inversions with endpoints proximal to the origin of DNA replication are nearly three times more frequent. Our findings represent the first characterization of genome arrangement evolution in a bacterial population evolving outside laboratory conditions. Insight into the process of genomic rearrangement may further the understanding of pathogen population dynamics and selection on the architecture of circular bacterial chromosomes.

## Author Summary

Whole-genome sequencing has revealed that organisms exhibit extreme variability in chromosome structure. One common type of chromosome structure variation is genome arrangement variation: changes in the ordering of genes on the chromosome. Not only do we find differences in genome arrangement across species, but in some organisms, members of the same species have radically different genome arrangements. We studied the evolution of genome arrangement in pathogenic bacteria from the genus *Yersinia*. The *Yersinia* exhibit substantial variation in genome arrangement both within and across species. We reconstructed the history of genome rearrangement by inversion in a group of eight *Yersinia*, and we statistically quantified the forces shaping their genome arrangement evolution. In particular, we discovered an excess of rearrangement activity near the origin of chromosomal replication and found evidence for a preferred configuration for the relative orientations of the origin and terminus of replication. We also found real inversions to be significantly shorter than expected. Finally, we discovered that no single reconstruction of inversion history is parsimonious with respect to the total number of inversion mutations, but on average, reconstructed genome arrangements favor “balanced” genomes—where the replication origin is positioned opposite the terminus on the circular chromosome.

## Introduction

Genome arrangement has profound effects on organismal phenotype. Genome arrangement likely impacts gene expression [1],[2],[3], and can result in total loss of gene function when a rearrangement breakpoint occurs inside a reading frame. Moreover, rearrangements are known to affect linkage and introduce genetic incompatibility in eukaryotes [4]. Similar recombination-stifling effects have been proposed in prokaryotes [5],[6], whose capacity for genetic exchange among divergent taxa has only recently been appreciated [7]. In naturally competent microbes which undergo frequent homologous recombination, genome arrangements themselves may be better indicators of vertical inheritance than other molecular characters.

Our ability to measure gene order and chromosome structure has undergone several revolutions, beginning with careful study of linkage maps [8], later moving towards direct observation by microscope, FISH, Radiation Hybrid, paired-end genome sequencing, and Optical Mapping techniques [9],[10],[11],[12]. The continued improvement in measurement technology has offered revelations regarding the pattern and extent of genome rearrangement in organisms ranging from bacteria [13] to mammals [14].

In circular bacterial chromosomes, DNA replication divides the circular chromosome into two domains called replichores. Replication begins when DNA polymerase holoenzymes anneal to the *origin of replication* (*ori*). Two holoenzymes then simultaneously copy the circular chromosome in opposite directions, and initially the DNA polymerase holoenzymes are co-localized in the cell in a so-called “replication factory” [15]. Each holoenzyme copies about half the chromosome, and they eventually meet each other in the *Ter macrodomain*. The *Ter macrodomain* spans a large portion of the chromosome opposite the origin of replication and contains several *ter* sites which bind proteins that halt procession of DNA polymerase [16]. In cases where homologous recombination has taken place during replication, the XerCD molecular machinery resolves the chromosome dimer at the *dif* site [17],[18]. Moreover, the predominant site of replication termination appears to be at or near the *dif* site [19]. We refer to each half of the chromosome, delineated by *ori* and *dif*, as a replichore. Hereafter we will use the word “terminus” or phrase “terminus of replication” to refer to the approximate location of the *dif* site.

Genome sequencing has revealed that rearrangements do not occur with uniformly distributed endpoints on circular prokaryotic chromosomes. Instead, a striking pattern of inversions with endpoints biased by the origin and terminus of replication has commonly been observed [20],[21],[22],[23]. Several explanations for the observed pattern have been devised, all of which focus on the nature of DNA replication in circular chromosomes.

An inter-replichore inversion refers to a chromosomal inversion with one endpoint in each replichore. Such inversions swap the relative orientations of the origin and terminus. If the inversion endpoints are equally distant from the origin, then replichore sizes remain unchanged—a so-called “symmetric inversion”. Previous genome analyses indicate that inversions typically occur with breakpoints in oppositely oriented repetitive elements [24],[25],[26]. When DNA damage occurs, the homology-dependent recombination-repair machinery recruits another copy of the repetitive element as a repair template. Inversions, deletions, and duplications occur when the resulting Holliday junction is incorrectly resolved. Whereas recombination among inverted repeats leads to inversions, recombination among direct repeats leads to deletion. When the recombination among direct repeats occurs during replication, the segment becomes deleted from one chromosome and duplicated in the other.

Bacterial DNA replication appears to induce a multitude of mutational biases and selective forces with respect to their chromosome architecture [27]. Chromosomes are thought to remain small due to a general deletion bias [28]. Strand-specific oligomers such as *χ* sites [29] assist with DNA repair, while KOPS/AIMS [30],[31] have roles in DNA replication and chromosome segregation. Such sequence signals would be disrupted by inversions within a single replichore, but not by inter-replichore inversions. Moreover, a large survey of *Salmonella* genomes in culture has provided evidence that genomes with equal-sized replichores (balanced replichores) may be under positive selection [32]. It is currently unknown whether symmetric inter-replichore inversions are frequently observed simply because they occur more frequently than other rearrangements (a recombination bias), or whether other patterns of rearrangement commonly occur but are strongly selected against [26].

The observed frequency of rearrangement relative to neutral substitution is highly variable in different organisms. The frequency of observed rearrangement in modern genomes correlates with the presence of repeats induced by mobile genetic elements [26],[33]. Interestingly, mobile genetic elements (IS elements/transposons) are also associated with the generation of pseudogenes, genome reduction, and adaptive evolution of niche change [34]. Large-scale inversion and deletion are both driven by homologous recombination among repeat elements. Taken together, these associations suggest that methods to predict episodes of ancient genome rearrangement may be able to uncover historical genome reduction and transitions in ecological niche.

Studies of *Yersinia* have revealed extensive genomic rearrangement relative to conspecific isolates, and IS elements have been implicated in the rearrangement process. The recent availability of several finished *Yersinia* genome sequences offers the possibility to investigate patterns and biases associated with genomic rearrangement. *Yersinia pestis* played a role as the causative agent of the three major plague epidemics which together resulted in millions of deaths over the past two millenia [35]. Previous molecular studies have indicated that *Yersinia pestis* is a recently emerged clone of *Y. pseudotuberculosis*, with an estimated divergence less than 20,000 years ago [36], although some ambiguity in the branching order of *Y. pestis* isolates remains [37].

Given its pathogenic lifestyle, *Y. pestis* population dynamics are different from those of non-pathogens and the effect of population dynamics on genome arrangement warrants consideration. Upon infection of a human host, *Y. pestis* likely undergoes expansive population growth. Transmission to a new host is usually mediated by a flea vector which can viably harbor only a small number of *Yersinia* cells compared to an infected human. As such, modern *Y. pestis* may have undergone several cycles of unconstrained population growth followed by extreme transmission bottlenecks. The unconstrained growth phase could permit generation of cell lines with genomic rearrangement, which are subsequently fixed by the transmission bottlenecks. Such population dynamics would serve to increase the observed rate of rearrangement.

Previous experimental work has characterized patterns of genome arrangement in isolates of *E. coli* and *Salmonella* whose genomes were artificially perturbed in the laboratory [38]. Our study represents the first attempt to quantify selection and recombination bias acting on genome arrangement in a naturally evolving population.

## Results

### Genome Arangement History of *Yersinia*

We apply a Bayesian MCMC sampler to investigate selection and recombination bias acting on genome rearrangements in sequenced *Yersinia* isolates. At the time of this study, nine finished *Yersinia* genomes were publicly available, listed in Table 1, and several more had been sequenced to draft quality. As the *Yersinia pestis* are very recently diverged, only a small number of nucleotide substitutions have been observed in fully sequenced genomes [39], and efforts to reconstruct the *Yersinia* phylogeny have consequently been forced to integrate presence/abscence patterns of IS elements and VNTR sequences [37].

**Table 1:** Fully sequenced *Yersinia* genomes analyzed for genome rearrangements.

Organism	Pathogenesis	Genome Size	*dif*	*o*	Accession	Ref
*Y. pestis* Antiqua	Plague	4,702,289 nt	0.39	+	CP000308	[39]
*Y. pestis* Nepal516	Plague	4,534,590 nt	0.43	+	CP000305	[39]
*Y. pestis* 15–70 (Pestoides F)	Plague	4,517,345 nt	0.77	+	NC009381	unpubl.
*Y. pestis* CO92	Plague	4,653,728 nt	0.55	+	AL590842	[54]
*Y. pestis* KIM	Plague	4,600,755 nt	0.51	+	AE009952	[25]
*Y. pestis* 91001	avirulent	4,595,065 nt	0.50	+	AE017042	[78]
*Y. pseudotuberculosis* IP 32954	enterocolitis	4,744,671 nt	0.54	+	BX936398	[79]
*Y. pseudotuberculosis* IP 31758	enterocolitis	4,721,828 nt	0.46	−	AAKT02000001	[80]
*Y. enterocolitica* 8081	enterocolitis	4,615,899 nt	0.48	+	AM286415	[42]

The reported genome size is the size of the primary circular chromosome without plasmids. The *dif* column indicates the approximate position of the replication terminus *dif* site, ranging between 0 and 1, where the origin of replication is at 0 and 1 on the circular chromosome. The *o* column indicates whether the origin and terminus *dif* site have the canonical relative orientation (+) or the inverse relative orientation (−): see text for details.

Pairwise comparisons of *Yersinia* genomes have revealed a large number of genomic rearrangements [25],[40] which may be suitable phylogenetic characters. As large-scale genome rearrangement is thought to be a low-homoplasy molecular character [41] impervious to lateral exchange by homologous recombination, even a small number of rearrangements may suffice to resolve phylogenetic tree topology.

### Genome Alignment and Replichore Sizes

In order to compute a rearrangement history, we require genomes to be encoded as a signed permutation matrix indicating order and orientation of homologous segments in each genome. We used the Mauve multiple genome alignment software to identify and align 84 Locally Collinear Blocks (LCBs) shared among the 9 *Yersinia* genomes. Differential gene content among *Yersinia* lineages precludes a nine-way alignment that completely covers each genome. On average 81.5% of each genome is contained within LCBs, and the remaining lineage-specific regions reside in breakpoint regions. The breakpoint regions cannot be unambiguously assigned to either neighboring LCB, and the uncertainty about their placement in ancestral genome arrangements causes corresponding uncertainty in ancestral replichore sizes.

While *Y. pestis* and *Y. pseudotuberculosis* share a majority of their gene content, *Y. enterocolitica* has substantial differential content relative to the other eight taxa [42]. To mitigate inference problems related to differential gene content (see Methods), we removed *Y. enterocolitica* from our analysis and computed an alignment on the remaining 8 taxa using a procedure described in Methods.

**Figure 1:**
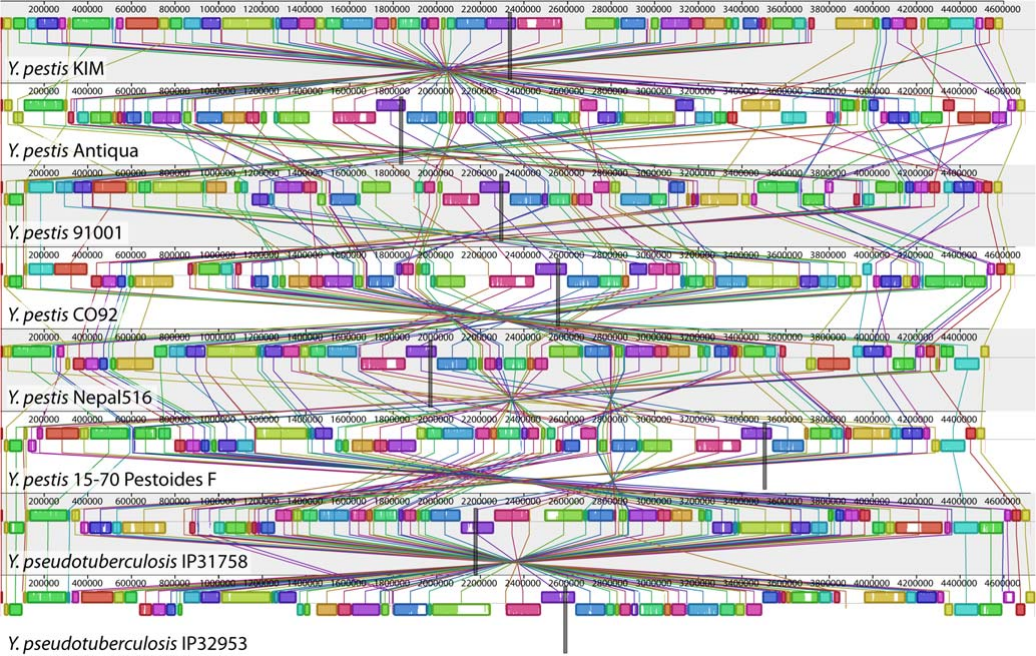
A genome alignment of eight *Yersinia* isolates. Whole genome alignment of eight *Yersinia* genomes using Mauve [77] reveals 78 locally collinear blocks conserved among all eight taxa. Each chromosome has been laid out horizontally and homologous blocks in each genome are shown as identically colored regions linked across genomes. Regions that are inverted relative to *Y. pestis* KIM are shifted below a genome's center axis. The origin of replication in each genome is approximately at coordinate 1 and the terminus *dif* sites are approximately midway through each genome, as marked by grey vertical bars. The termini were identified by sequence comparison with *Y. pestis* KIM, where they were characterized by extensive sequence analysis [25]. Figure generated by Mauve, free/open-source software available from http://gel.ahabs.wisc.edu/mauve.

**Figure 2:**
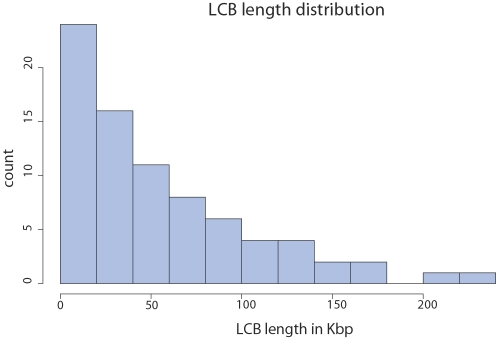
Lengths of Locally Collinear Blocks shared by the eight *Yersinia* genomes. Block lengths are taken from the *Y. pestis* KIM reference genome.

The alignment of eight *Y. pestis* and *Y. pseudotuberculosis* strains, shown in Figure 1, consists of 78 LCBs (79 before considering genome circularity) that cover an average of 93.3% of each genome. The distribution of LCB lengths (Figure 2) appears to be geometric, consistent with expectation under the Nadeau-Taylor random breakage model [14]. For the purpose of inferring ancestral replichore sizes, we divide each of the 78 breakpoint regions in half and assign each half to a neighboring LCB. The origin and terminus of replication in each genome were assigned on the basis of a consensus prediction and homology (see Methods).

### Bayesian Analysis of Rearrangement Phylogeny

We used a modified version of the BADGER 1.01b software to sample the posterior probability distribution of phylogenetic trees, mutation rate, and genome arrangement histories using inversions as mutation operations. The model treats all inversion events to be equally likely *a priori*, with no explicit preference for rearrangements that maintain or improve replichore balance. The prior distribution on branch lengths creates a strong preference for histories with fewer inversions. Like other Bayesian MCMC samplers for phylogenetics, the method used here creates an initial phylogenetic tree with mutation events mapped onto the branches, then repeatedly proposes modifications to the current tree topology, mutation history, and branch lengths. Any proposed modifications are accepted with probability dictated by the Metropolis-Hastings ratio [43],[44]. The initial proposed reconstruction of inversion history typically has very low likelihood and proposed modifications are generally accepted until the likelihood reaches a local maxima. The initial period of sampling is commonly referred to as *burn-in*. Samples taken during burn-in are discarded since the Markov-chain has not yet converged to the true posterior distribution.

**Figure 3:**
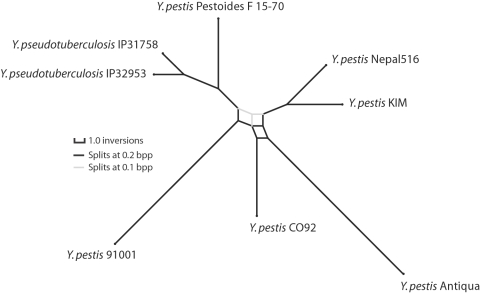
Consensus phylogenetic network of *Yersinia* based on inversions. Consensus phylogenetic network for eight of the *Yersinia* listed in Table 1. Branch lengths are proportional to the average number of per-branch inversion events. Splits with Bayesian posterior probability (Bpp)>0.2 are shown in black, splits with Bpp between 0.1 and 0.2 in gray. To visualize the network at Bpp 0.2, imagine removing gray edges and straightening the black edges. The inversion phylogeny supports a *Y. pestis* clade, and at Bpp 0.2 it supports subclades which agree with SNP phylogenies [39]. Of note, internal branches in the *Y. pestis* are short relative to *Y. pseudotuberculosis*, suggesting either rapid population growth, subdivision, or other effects. Network visualization created using SplitsTree 4 [45].

As applied to the 78 *Yersinia* LCBs, we ran chains with 1,510,000 modification proposal steps, discarded the first 10,000 steps of each chain as burn-in and then subsampled every 50 steps (details in Methods). The resulting posterior sampling consists of 30,000 complete genome arrangement histories. Each sampled history contains a tree topology with inversion events mapped onto the branches. In total, the sampled histories contain 30,000 tree topology estimates and 2,520,185 genome arrangements, of which 2,280,185 are inferred ancestral arrangements and 240,000 are modern genome arrangements. Visualization of the posterior distribution of trees using SplitsTree v4 [45] reveals a small amount of topological ambiguity as a splits network (Figure 3). Contributing to topological ambiguity are seven different tree topologies with parsimonious inversion histories of 79 events. All seven parsimonious topologies differ in their grouping of *Y. pestis* isolates. Nonetheless, the *Y. pestis* are found to be monophyletic, with subgroupings that are consistent with previously published genome analyses [39]. Application of a maximum parsimony algorithm to reconstruct inversion phylogeny recovers one of the seven parsimonious topologies identified by BADGER, also with 79 inversions [46],[47]. Internal branches of the *Y. pestis* clade are very short relative to external branches, a phenomenon which could have numerous explanations including exponential population growth, population subdivision, an ancestral selective sweep, or recently accelerated mutation rates possibly associated with pathogen population dynamics or relaxed selection in culture. Of note, SNP phylogenies also exhibit short internal branches [39].

### Visualizing Inversion History

To quickly scan for patterns in the genome rearrangement history of *Yersinia*, we developed a 3D video system to visualize the series of rearrangement events. The posterior sampling of inversion history contains 30,000 samples. We selected the one history with maximum *a posteriori* probability and rendered the series of rearrangement events on each branch of the phylogeny using custom Java software. The chromosome is rendered as a torus with positions of the replication origin and terminus marked. The replichores present in an ancestral node of the tree are colored distinctively, left replichore in blue, right replichore in green. The intensity of the colors changes on a gradient from origin to terminus, such that segments near the origin in the ancestor are dark blue or green, while segments near the terminus are light.

Supplementary Videos S1, S2, S3, S4, S5, S6, S7, and S8 show the inversion history along each external branch of the maximum *a posteriori* tree estimate. Several striking patterns of rearrangement can be seen in the videos, especially those representing longer branches such as the branch leading to *Y. pestis* 91001 (Video S3). First, the terminus remains positioned mostly opposite the origin throughout the rearrangement history. Second, light-colored segments which were near the terminus in the ancestral genome arrangement tend to remain near the terminus. Third, when large inversions happen within a single replichore, they appear to be quickly followed by a second inversion that reverts the first. We now describe statistics to quantify the significance of these observations, along with other aspects of genome arrangement evolution that are not as easily recognizable through visualization.

### Selection for Replichore Balance

When the terminus of replication lies opposite the origin on the circular chromosome, replichore sizes are equal and the genome is said to be balanced. If we assume the origin is at positions 0 and 1 on the circular chromosome and the terminus *dif* site lies at some position *b* where 0<*b*<1, we can quantify the degree of imbalance as the deviation from perfect balance: 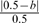. Thus, a perfectly balanced genome with *b* = *0.5* will have 0 imbalance, and imbalance increases to 1 as the terminus *dif* site position *b* approaches 0 or 1.

**Figure 4:**
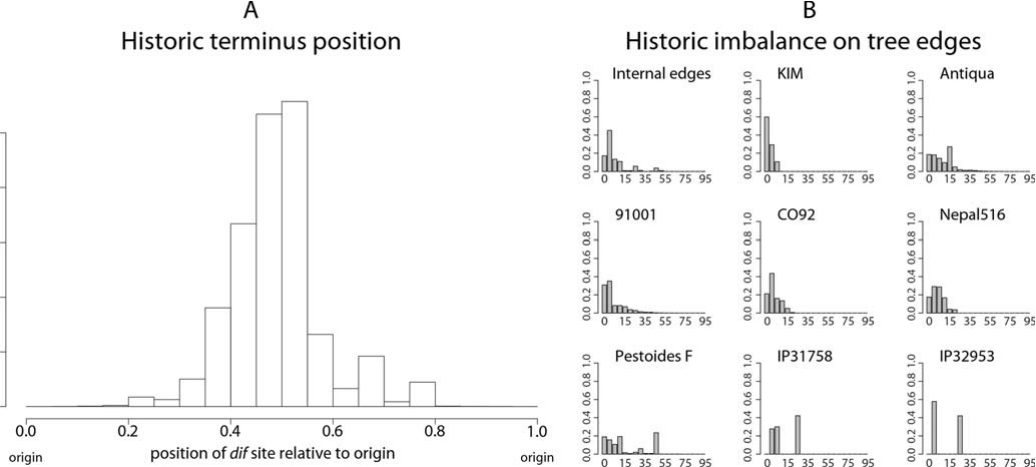
Historic replichore balance in *Yersinia*. Historic position of terminus *dif* site relative to origin (A) and historic degree of imbalance (B) observed in all sampled ancestral genome arrangements of the eight *Yersinia* listed in Table 1. The histogram in (A) shows the replichore balance of all sampled ancestral and extant genome arrangements of the *Yersinia*. In (A) an arrangement with equal replichore size will have a terminus at position 0.5, indicating perfect replichore balance. The diagram shows that >88% of sampled genome arrangements have replichores within 30% of perfect balance. (B): Histograms showing the degree of imbalance for arrangements sampled on branches leading to modern genomes. Each histogram is labeled with the corresponding strain's name. Genomes with perfectly balanced replichores have 0% imbalance while a genome with the origin and terminus at the same locus would have 100% imbalance. Many, but not all, parsimonious inversion histories have imbalanced genome arrangements at common ancestors of *Y. pseudotuberculosis* and *Y. pestis* Pestoides F that contribute toward the observed imbalance in the posterior distribution for those taxa.

Of the 2,520,185 sampled ancestral arrangements, 77.9% of the arrangements have a replichore within 20% of perfect balance, and 88.5% are within 30% of perfect balance. The full distribution of balance for ancestral arrangements can be gleaned from the historic terminus position plot in Figure 4A. To prove that the ancestral positioning of the terminus can not be explained by a series of inversions with arbitrary endpoints, we performed 30,000 simulations of replichore balance evolution in a genome that undergoes inversions with uniformly chosen endpoints. Comparison with the null model suggests it can not explain the observed data (KS test, median *p*-value<10^−1^). Even when the simulated terminus *dif* site position is restricted to the range observed in modern genomes, the null model cannot explain the observed genomic balance (KS test, median *p*-value≈0.0001).

Not all modern genomes are balanced genomes. *Y. pestis* Pestoides F is conspicuously imbalanced, with a terminus position of 0.77 (54% imbalance). As such, we might ask whether the imbalance observed in ancestral genome arrangements is confined to the *Y. pestis* Pestoides F lineage. Figure 4B shows the imbalance observed on each external branch of the phylogeny, with internal branches pooled. Clearly all lineages undergo imbalance, although the Pestoides F isolate has a greater fraction of imbalanced genomes in its history. Surprisingly, the *Y. pseudotuberculosis* exhibit a high degree of imbalance as well. As they are sister taxa to Pestoides F, the imbalance could be attributed to imbalance at the common ancestor. In fact, the common ancestor is frequently predicted to have an imbalanced genome, and reconstructions with a balanced common ancestor require intermediate states of imbalance on branches leading to the modern *Y. psuedotuberculosis* genomes.

Alternative explanations for the unusual terminus position in *Y. pestis* Pestoides F could be entertained, one such explanation being assembly error. As the assembly has been validated using a 40 kb Fosmid library, we do not believe this to be the case (P. Chain, personal comm.). Another alternative is that the primary replication terminus has shifted to a different location in the *Y. pestis* Pestoides F lineage. Visual inspection of the rearrangement pattern for *Y. pestis* Pestoides F in Figure 1 reveals several instances of local overlapping inversions characteristic of symmetric inversion about the terminus (seen as a “fan” pattern of crossing lines). If Pestoides F has indeed switched to a new primary terminus site it would introduce some error in our calculation of the historic replichore balance distribution. However, since only about 10% of inversions occur on the branch leading to *Y. pestis* Pestoides F, the error would be negligible. The error would serve to overdisperse the estimated balance distribution and result in weaker apparent bias towards replichore balance.

Substantial ambiguity exists in the phylogenetic tree topology reconstructed from the *Yersinia* genome arrangements. BADGER found seven parsimonious topologies, and in total 48 unique topologies were sampled with inversion counts ranging from 79 to 87. Parsimony has enjoyed a long history as a guiding philosophy in evolutionary inference, so it is of interest to know whether parsimonious reconstructions agree with our expectation of replichore balance in genome arrangements. The mean estimate of imbalance turns out to be slightly smaller for parsimonious histories and the variance is much lower, as shown in Table 2. The difference in balance between parsimonious and other reconstructions is significant (KS test, *p*<2e-16) but the difference is small (KS D = 0.016). If we believe that strong selection for balanced genomes exists and inversions not affecting balance are neutral, then parsimonious reconstructions appear slightly more favorable.

**Table 2:** Degree of imbalance as a function of total number of inversions.

# inv	79	80	81	82	83	84	85	86	87
B. mean	0.128	0.133	0.135	0.137	0.139	0.144	0.143	0.149	0.156
B. sd	0.115	0.122	0.125	0.128	0.131	0.139	0.133	0.142	0.135
KS *p*	<2e-16	<2e-16	2e-5	0.02	0.008	0.18	0.22	0.27	-
KS D	0.016	0.010	0.007	0.008	0.017	0.020	0.037	0.105	-
N	11492	11395	4775	1661	498	130	38	10	1
Bpp	0.383	0.379	0.159	0.055	0.017	0.004	0.001	<0.001	<0.001

The posterior estimate of the mean degree of imbalance (B. mean) and associated standard deviations (B. sd) are given for inversion histories of length ranging from 79 to 87 (# inv). For each successive pair of inversion counts, the distribution of balance values for genomic arrangements was compared using a Kolmogorov-Smirnov (KS) test, with *p*-values and D-values reported as KS *p* and KS D. N gives the number of samples and Bpp gives the total amount of Bayesian posterior probability for each inversion history length. From the data we conclude that parsimonious histories (79 events) have better-balanced genome arrangements, but the difference is small (KS D) even though it is statistically significant.

### Symmetric Inversions

Previous studies have suggested that DNA replication introduces a recombination bias that favors inversions with endpoints that are equally distant from the origin of replication [22],[20], so-called symmetric inversions. Given our inferred inversion histories, we can formally test for an excess of symmetric inversions. To do so, we introduce the following notation. Let *V* be the ordered set of inversions mapped onto tree branches in a sampled reconstruction of the inversion history, and let *v_i_* represent the *i^th^* inversion. Then we define a symmetry statistic for inter-replichore inversions as:
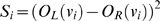
(1)where *O_L_*(*v_i_*) is the distance between the origin and the left-end of the *i^th^* inter-replichore inversion, while *O_L_*(*v_i_*) is the distance between the origin and the inversion's right-end. Thus, the equation expresses the distance between inversion endpoints and the origin in each replichore, and computes the squared-difference of distances. Equation 1 assigns a perfectly symmetric inversion a value of zero, while asymmetric inversions take on large values. Incidentally, the symmetry statistic is agnostic to the choice of which replichore is the left or right.

We would like to know whether the observed inversions are more symmetric than expected by chance. To do so, we use permutation to generate a distribution of symmetry statistics that represent the null hypothesis of lack of symmetry. We compute the symmetry statistic on arbitrary pairs of left and right inversion endpoints from inter-replichore inversions, according to the following equation:
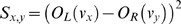
(2)


More formally, we compute a null distribution by sampling *x* and *y* uniformly without replacement from the set of possible inter-replichore inversions. *O_L_*(*v_x_*) represents the distance from the origin to the left-side of inversion *x*, and *O_R_*(*v_y_*) is the distance from the origin to the right-side of inversion *y*. If the inversion endpoints on the two replichores were independent from each other, then we would not see a significant deviation from the null distribution. Deviation towards larger values would imply fewer symmetric inversions than expected, whereas deviation towards smaller values implies more symmetric inversions than expected.

**Figure 5:**
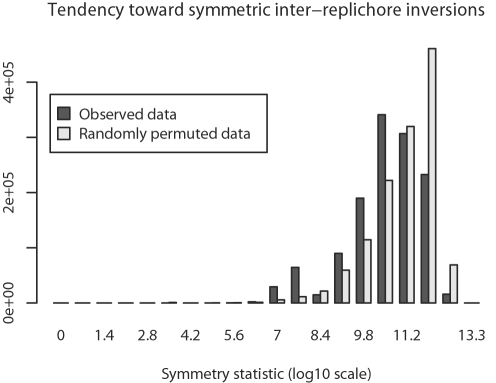
Inter-replichore inversions exhibit symmetry. Inter-replichore inversions exhibit greater symmetry about the origin and terminus than expected under a null model. Symmetry for inter-replichore inversions has been quantified by Equation 1 and compared to a null distribution. The null distribution is created by applying the permutation statistic in Eqn 2 to each of the 30,000 sampled rearrangement histories. The pooled posterior samples and permutations are plotted here, statistical tests are done on a per sample basis.

Comparison of symmetry statistics generated by Equations 1 and 2 demonstrates that within-replichore inversions are more likely to be symmetric than expected by chance (KS test, median *p* = 0.0001, mean D = 0.47). The observed symmetry statistic distribution and the corresponding null distribution are shown in Figure 5.

### Episodes of Imbalance

**Figure 6:**
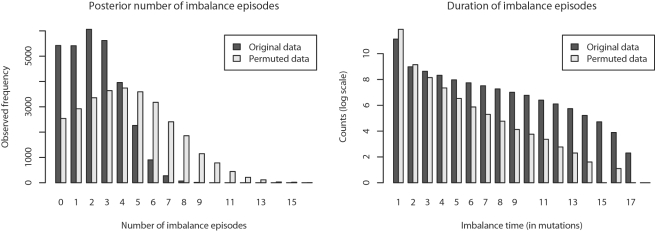
Episodes of imbalance in *Yersinia*. Left: The Bayesian posterior distribution of the number of imbalance episodes occurring entirely on branches of reconstructed inversion phylogenies, compared with permuted data. Right: Posterior distribution of the imbalance episode duration (in mutation events) observed on branches, compared with data permuted as described in the text. From the two plots we can conclude that transitions to imbalance are less frequent than expected under a null model, and that imbalance episodes last longer than expected under the null model.

Our inference method does not estimate event times but only relative event ordering, thus we are unable to directly infer the actual amount of time ancestral genomes have spent in a balanced state. However, if we define a state of imbalance as a percentage deviation from perfect balance, say a 20% deviation, then we can quantify the number of imbalance episodes that the organisms have undergone. The posterior estimate of the number of imbalance episodes the eight *Yersinia* have undergone is 3.26 (*σ* = 1.82), not counting episodes which span a bifurcation event in the tree. The posterior distribution is shown at left in Figure 6. Similarly, we can define the duration of an imbalance episode as the number of mutation events (inversions) experienced before the chromosome returns to a balanced state. The length of imbalance episodes observed in our posterior sampling is shown at right in Figure 6.

If imbalance is strongly selected against, we might expect episodes of imbalance to be very short and more frequent than expected by chance given the total number of imbalanced arrangements. To determine whether the number and duration of imbalance episodes was unusual, we designed a permutation test in which the balance states along branches of reconstructed trees were randomly permuted (see the Methods section for details). The permutation gives a null model of an organism which freely transitions to and from balance, spending the same total amount of time in each state as the *Yersinia* genomes.

Surprisingly, we find the exact opposite of our initial expectation. There are fewer imbalance episodes than expected under the null model, and episodes of imbalance are longer than expected given the null model. The pattern is robust to choice of a particular balance threshold, as other thresholds up to 40% give similar results. Explanations might be that imbalance is only mildly detrimental, or that transmission bottlenecks periodically fix suboptimal genome arrangements in lineages of *Y. pestis*, despite their fitness disadvantage. Once imbalanced, several inversions typically occur before balance is restored. Given that the *Y. pestis* chromosome is littered with repetitive DNA, the observation is consistent with the notion that picking an arbitrary pair of oppositely oriented repeats is unlikely to yield an inversion that restores balance. Under such a hypothesis, the number of inversions occurring before restoration of balance should rise with the frequency of oppositely oriented repetitive DNA.

### Inversion Length

**Figure 7:**
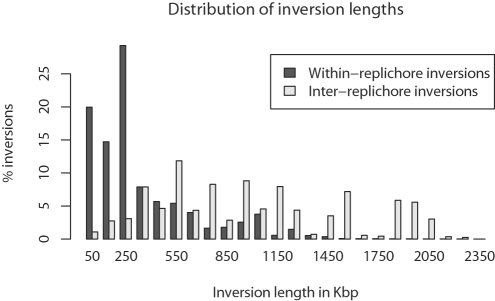
The posterior distribution of inversion lengths in *Yersinia*. Inversions have been classified as inter-replichore (those which span the origin) and within-replichore. The observed within-replichore inversions have a strong tendency to be short, whereas the inter-replichore inversions have a more uniform length distribution.

Assuming that no selection or recombination bias acts on inversion length, the distribution of inversion lengths could be modeled as the distance between two uniformly chosen points on a circle with circumference 1. However, 46.3% of sampled inversions act within a single replichore and we might expect such inversions to be short relative to inter-replichore inversions. Although they do not affect balance, inversions within a replichore act to reverse the polarity of *x* sites [29], KOPS/AIMS [30],[31], and they also change leading/lagging strand A/T and G/C biases [48], relative gene density [27], and gene expression levels. As shown in Figure 7, the observed length distribution for within-replichore inversions does indeed indicate that they are shorter than inter-replichore inversions. However, we expect inter-replichore inversions to be longer than within-replichore by definition, because inter-replichore inversions must have one endpoint in each replichore.

To determine whether within-replichore inversions are significantly shorter than inter-replichore inversions, we develop a null model of inversion length that accounts for replichores. Replichore sizes change as the position of the terminus *dif* site changes over the course of evolution, thus the expected length of within-replichore and inter-replichore inversions changes. We assume that inversion endpoints are uniformly distributed and that no inversion acts on more than half the chromosome, otherwise a shorter complementary inversion operates on the other side of the circular chromosome. We can then define the expected length of a within-replichore inversion as:
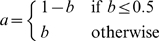
(3)

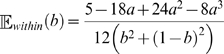
(4)where 0<*b*<1 is the position of the terminus *dif* site relative to the origin of replication. We define a similar measure of expected length for inter-replichore inversions:
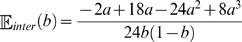
(5)


**Figure 8:**
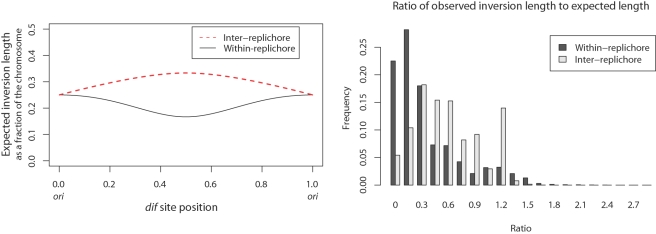
Inversions are shorter than expected. Left: The expected length of within-replichore and inter-replichore inversions assuming that inversion endpoints are uniformly distributed. The expected length changes as a function of the positioning of the terminus *dif* site relative to the origin of replication. In general, within-replichore inversions are expected to be shorter than inter-replichore inversions. Right: The ratio of observed inversion length to expected length for all sampled within- and inter-replichore inversions. Both inter- and within-replichore inversions are shorter than expected, but within-replichore inversions are much more so than inter-replichore inversions.

We provide a detailed derivation of these equations in the Methods section, and the values given by each equation for 0<*b*<1 are shown at left in Figure 8.

Knowing the expected length for each inversion, we compute the ratio of observed length to expected length for each inversion in the posterior sampling. The distribution of ratios for within- and inter-replichore inversions is given at right in Figure 8. Both classes of inversion are shorter than would be expected under the null model. Comparison among within- and inter-replichore inversions reveals that within-replichore inversions are much more so than inter-replichore inversions (KS test, median *p* = 0.002, mean D = 0.41).

### Selection on the Orientation of *ori* and *dif*

Previous study of *Salmonella* isolates has demonstrated that inversion of the origin relative to the terminus does not have a noticeable fitness impact, so long as balance is maintained [32]. Despite that, eight of the nine *Yersinia* genomes have the origin and terminus in identical relative orientation, which we term the canonical OriDif configuration (see Table 1). The configuration can be readily observed in Figure 1 by noticing that blocks containing the *dif* site (purple) are shifted upwards in every genome except *Y. pseudotuberculosis* IP31758, as are blocks containing the origin (extreme left and right in Figure 1). If the canonical OriDif offers no selective advantage over the non-canonical configuration, then observation of the canonical OriDif can be modeled with a binomial distribution. Under the binomial, the probability of observing eight of nine genomes with the canonical OriDif is 0.018, suggesting that a preference for the canonical OriDif configuration must exist. The genomes of *Y. pestis* Angola and *Y. pseudotuberculosis* YPIII were finished while this manuscript was under review and they too exhibit the canonical OriDif configuration, bringing the tally to 10/11 and *p*<0.01. Of note, studies of mutation patterns in diverse bacteria suggest that replication terminates near the *dif* site itself, despite the presence of many additional *ter* sites [19]. Although it is tempting to generalize the canonical OriDif idea to other bacterial genomes, a cursory examination of related heavily rearranged *Shigella* genomes did not reveal a preference for a canonical OriDif configuration.

That modern isolates favor the canonical OriDif configuration suggests that ancestral *Yersinia* would favor it as well, and probably also spend a noticeably greater amount of time in such a configuration. Most genome rearrangements in *Yersinia* (53.7%) are inter-replichore inversions which swap canonical and non-canonical OriDif configurations. As such, the number of arrangements with the canonical OriDif is not substantially different from those which have the non-canonical arrangement.

**Figure 9:**
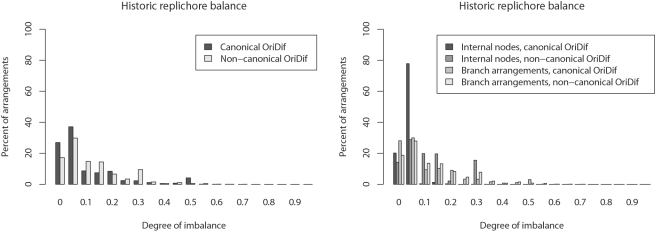
Association between replichore balance and the relative orientation of *ori* and *dif*. Left: Balance for canonical and non-canonical OriDif configurations. Right: Balance as a function of whether arrangements are at an internal node or along a branch. Arrangements at internal nodes of the phylogeny appear to be better balanced, but only when *ori* and *dif* are in the canonical orientation.

Given that modern genomes tend towards balance and a canonical OriDif, we might expect an association between balance and OriDif because an inversion that disrupts balance must also change the relative orientation of the origin and terminus. The left panel of Figure 9 shows overall balance of arrangements as a function of OriDif configuration. A significant association between balance and canonical OriDif can be seen (KS test, median *p* = 0.0015, mean D = 0.4). Interestingly, when arrangements at internal nodes of the phylogeny are compared to branch arrangements, the association between canonical OriDif and balance appears to be more pronounced (Figure 9 right). However, a comparison of balance at internal node arrangements with canonical OriDif versus branch arrangements with canonical OriDif fails to demonstrate a significant difference (KS test, median *p* = 0.67, mean D = 0.33). Failure to find a significant difference may be due to lack of inferential power, since each inversion history sample has only six internal node arrangements from which to estimate the balance distribution. Additional finished *Yersinia* genome sequences would provide greater statistical power.

### Hotspots of Rearrangement

The most-parsimonious inversion histories inferred by BADGER contain 79 inversion events, yet only 78 gene-order breakpoints exist in the *Yersinia* genomes. Clearly, some breakpoints must be used repeatedly. Previous breakpoint re-use studies [49],[50] have typically relied on inferring the mere existence of reuse rather than identifying rearrangement hotspots. To do so, we must shift focus from breakpoints to inversion endpoints. Every inversion event acts to reverse one or more consecutive LCBs. The left side of the left-most and right side of the right-most reversed LCBs constitute the inversion endpoints. As such, we can count the number of times a given LCB boundary is used in an inversion history. By definition, every LCB boundary must be the endpoint of at least one inversion, however some LCB boundaries may be used more than once.

**Figure 10:**
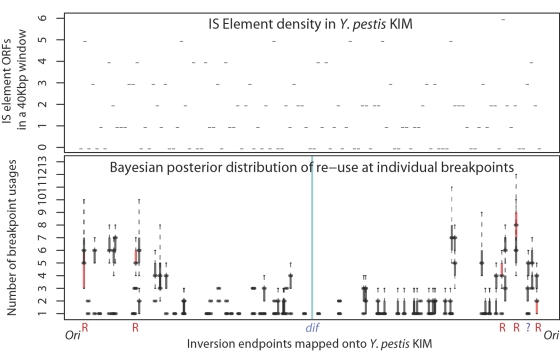
Hotspots of breakpoint re-use in *Yersinia* exist near the origin. Top: Number of annotated IS element ORFs in non-overlapping 40 Kbp windows of the *Y. pestis* KIM genome. Bottom: Hotspots of breakpoint re-use in *Yersinia*. The 78 blocks have 156 endpoints. Posterior estimates of the number of times each endpoint has been used are plotted here, with block endpoints positioned according their location in the *Y. pestis* KIM genome. Endpoints within 1500 bp of a ribosomal operon in at least one of the eight genomes are colored red and marked by ‘R’, while endpoint regions containing an annotated IS element are colored black. Only one breakpoint region is free from IS elements and ribosomal genes in all genomes, as marked by ‘?’. Together, the top and bottom panels demonstrate that we rarely observe inversions with endpoints proximal to the terminus in *Yersinia*, despite the presence of numerous IS elements in that region.

Figure 10 shows the posterior estimate of usage for individual LCB boundaries, mapped according to their occurrence in the *Yersinia pestis* KIM genome. A striking pattern emerges in which inversion endpoints lie proximal to the origin of replication much more frequently than to the terminus. While inversions with endpoints near the terminus of replication do occur, they are comparatively rare.

Experimental studies of genome rearrangement in *E. coli* and *Salmonella* have pointed towards the existence of chromosomal domains near the terminus that can not tolerate inversion endpoints [38], termed the “impermissible zones”, or “non-divisible zones”. *Yersinia* appear to have a similar constraint, visible as the region immediately surrounding *dif* having 0 or 1 inversion endpoints. An alternative and very plausible explanation is the presence of AIMS proximal to the terminus of replication [31]. AIMS are polarized motifs that direct chromosomal segregation during cell division, and the density of such motifs increases with proximity to the terminus *dif* site. Reversal of a large AIMS-rich segment could severely disrupt chromosome segregation.

In other Enterobacteriacae, frequent chromosomal inversion has been attributed to the presence of rRNA operons proximal to the origin [51]; as they are conserved in sequence, these operons provide a large substrate for homologous recombination. To investigate whether ribosomal RNA operons were involved in the large number of observed rearrangements we assessed the presence of rRNA operons in modern isolates. In Figure 10, inversion endpoints which have an annotated ribosomal RNA gene within 1500 bp of the endpoint have been highlighted red and marked with R. Although the most commonly used inversion endpoint does border a ribosomal operon, the majority of heavily used endpoints do not. Instead, all but one of the remaining inversion endpoints have an annotated transposase or IS element ORF within 1500 bp. Thus the difference in observed inversion rate among ribosomal operons and transposable elements is not appreciable.

If inversions with endpoints near the terminus are forbidden, then the relative terminus position has limited range with respect to the origin. Thus, we might revisit the question of whether the observed replichore balance distribution can be explained by a neutral model of inversion. As with the unconstrained model, simulations of replichore balance evolution which restrict the relative terminus position to the range of [0.25,0.75] fail to explain the observed distribution of replichore balance (KS test, median *p*-value = 0.0001).

### Inversion Reversions

The Bayesian posterior distribution of the terminus position (Fig. 4A) shows that replichore balance has been largely maintained during the evolution of *Yersinia* genomes. To demonstrate that the observed pattern does not result from inversion followed by an immediate reversion with approximately the same endpoints, we introduce the following statistics. As above, let *V* be the ordered set of inversions for all edges in the tree and let *v_i_* refer to the *i^th^* inversion. We refer to the left endpoint of inversion *v_i_* as *L*(*v_i_*) and the right endpoint as *R*(*v_i_*). Note that genome coordinates range from 0 to 1, so that 0≤*L*(*v_i_*)≤*R*(*v_i_*)≤1. We compute the following statistic for consecutive pairs of inversions *v_i_* and *v_i_*_+1_:
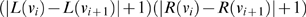
(6)


The value in Equation 6 is smallest when consecutive inversions have identical endpoints, in which case the second inversion effectively reverts the first inversion. However, since our Bayesian model of genome rearrangement favors histories with fewer overall inversions, it will only very rarely sample histories that contain consecutive inversions that perfectly cancel each other out. It will, however, sample consecutive inversions with nearby endpoints in an unbiased manner. Such a pattern of inversion could be caused by an unknown mutational or selective force that favors immediate reversion of inversions, such as a rebalancing inversion.

**Figure 11:**
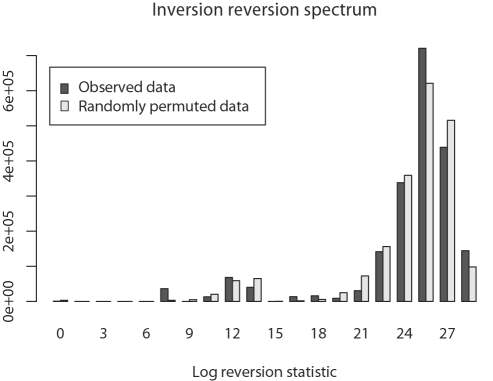
Testing whether inversions are immediately reverted by a second inversion with approximately identical endpoints. Shown is the distribution of statistics described in Equation 6 for consecutive inversions in the posterior distribution of inversion histories (dark gray) and null expectation by randomly paired endpoint distances (light gray). If selection or a recombination bias favoring immediate reversion of imbalanced replichores explains the tendency towards balance, we would expect to see consecutive inversions sharing approximately equal endpoints more frequently than by chance alone. The difference between observation and null expectation is not significant (see text).

Figure 11 compares the observed distribution for Equation 6 to a permuted distribution generated by pairing *L*(*v_i_*)−*L*(*v_i_*_+1_) values with *R*(*v_j_*)−*R*(*j_i_*_+1_) for *i*, *j* sampled uniformly without replacement. The observed distribution appears to be very similar to the permuted distribution. The difference is not significant (KS test, median *p* = 0.86, mean D = 0.1), indicating that consecutive inversions with nearly equal endpoints are not observed more frequently than would be expected by chance alone.

## Discussion

Genome rearrangement is a universal process in prokaryotes [20],[22], many of which exhibit patterns of rearrangement similar to that observed in *Yersinia*. Whereas previous studies have identified patterns of rearrangement in a laboratory setting, ours is the first detailed statistical study of such pressures in a naturally occurring population. *Yersinia* genomes provide an ideal platform for such a study, as they have recently diverged and have undergone little gene flux.

### Natural Selection versus Recombination Bias

We have identified several inversion patterns which deviate substantially from null expectation that all inversions are equally likely. Do our observations result from selection against some inversions, or is there a recombination bias which causes some inversions to occur more frequently than others? Our statistics can not directly quantify the relative contributions of these two evolutionary forces.

We might argue that balanced replichores result from weak-to-moderate positive selection. Our observation that episodes of imbalance are less-common than expected and last longer than expected could indicate that in general, imbalance is selected against, but when it occurs it is only mildly deleterious because balance is usually not immediately restored. Occasional relaxed selection on balance could be a function of pathogen population dynamics. On the other hand, a similar pattern could be induced by a recombination bias which usually preferred inversions with endpoints equidistant from the origin. Imbalance would be occasionally introduced by an inversion with endpoints of unequal distance from the origin, and because rebalancing requires a second inversion with endpoints of unequal distance from the origin, it may take many inversions to restore balance.

Our observation that *Yersinia* has a canonical OriDif configuration seems most easily explained by natural selection. A recombination bias introducing such a pattern would have to cause inter-replichore inversions to occur almost exclusively in pairs, and to our knowledge, no plausible molecular mechanism has been described which could achieve such a feat. Incidentally, if the canonical OriDif results from selection it implies that some symmetric inversions may be mildly deleterious in *Yersinia*.

Our observation that inversions with endpoints near the terminus are much less frequent than inversions with endpoints near the origin could be explained by selection against such inversions. If *Yersinia* is under reduced selection for growth rate, it may be more tolerant of inversions near the origin. Closely related organisms such as *E. coli* are known to have several *ter* binding sites throughout the half of the chromosome surrounding the terminus *dif* site. The *ter* sites are polarized motifs, such that they halt replisome procession only in one direction [16]. As such, a within-replichore inversion involving a *ter* site may result in a lethal disruption of DNA replication. A similar deleterious effect could be envisioned when inverting AIMS-rich segments.

We might also entertain recombination bias as an explanation for the excess of inversions with endpoints near the origin. Fast-growing bacteria are known to have multiple replication forks [52]. If the regions near the origin of replication exist in higher copy number they may be more prone to rearrangement, but higher copy number would also result in higher effective population size (*N_e_*) which might be expected to counteract the effect of a higher mutation rate. In any case, Figure 10 exhibits a precipitous shift from high inversion rate to low rate moving away from the origin. Although a plausible mechanism exists for selection against within-replichore inversions proximal to the terminus, the reasoning does not apply to inter-replichore inversions, which account for over half of all inversions. Given that the rate of inversion is about three times higher near the origin, it seems likely that additional unknown forces of recombination bias or selection play a role in reducing the inversion rate near the terminus.

### Arrangements as Phylogenetic Characters

Accurate genome arrangement phylogenies have the potential to provide a reference phylogenetic tree topology against which hypotheses of recombination, gene conversion, and lateral gene transfer can be tested. Chaisson *et al* [53] demonstrated that carefully filtered mammalian microinversion markers could be used as binary characters that form a perfect phylogeny, and a similar approach could be envisioned for microbes. Although Chaisson *et al* claim that rearrangements are low-homoplasy characters based on the ability of their (carefully filtered) data to pass the four-gamete test, three confounding factors stymie such simple approaches to rearrangement phylogeny when studying complete genome arrangements. First, rearrangement mutations frequently overlap each other, creating inter-dependence and thus precluding a clear representation of mutations as binary characters. Second, population-level variability in genome arrangement has been reported in both microbes [54] and mammals [55], implying that lineage-sorting effects may yield genome arrangement trees that do not match the species tree. Finally, programmatic rearrangement [56],[57] not only introduces population-level variability, but can repeatedly invert the same chromosomal segment, potentially resulting in frequent homoplasy.

It should be emphasized that PCR-based assays have identified mixtures of genome arrangements in laboratory cultures of *Y. pestis* [54]. If genome rearrangements such as symmetric inversions are nearly-neutral mutations, we would expect their frequency in the population to approximately follow a Wright-Fisher model. Thus, populations with a high rearrangement rate are likely to have more than one genome arrangement present. To our knowledge, no evidence of programmatic rearrangement mutations in *Y. pestis* has been reported that would be likely to cause frequent reversion and homoplasy in large-scale rearrangement mutations. Such effects have been observed as part of phase variation in other microbes [56].

### Related Work

Whilst rich stochastic models of nucleotide sequence evolution have been developed, comparatively little effort has gone into development of stochastic models of genome arrangement evolution. Inversions are known to affect a variety of genomes, including mitochondria [58], plastids [59],[60] and bacteria. However, mutational processes such as transposition or segmental duplication and loss [61] can also result in genomic rearrangement, and can have an especially profound effect on eukaryotic and mitochondrial gene order. Future efforts to model genome arrangement evolution should undoubtedly address duplication/loss.

Although bacteria are usually unichromosomal, they also have plasmids and other short circular chromosomes that might play an important role in rearranging the genetic material. Therefore a Bayesian MCMC method for multichromosomal genome arrangement phylogeny would also be desirable. Pairwise models of multi-chromosomal rearrangement via circular intermediates have recently been derived, although not in a Bayesian context [62],[63],[64].

The rearrangement patterns inferred by our study should prove valuable as a guide for phylogenetic inference when the inversion history signal has become saturated. The *Yersinia* genomes studied here appear to lie precisely on the verge of saturation, as seven parsimonious topologies were discovered. Just as codon models and gamma-distributed rate heterogeneity have aided phylogenetic inference on nucleotides, models of rearrangement which explicitly acknowledge that not all genome arrangements are equally likely may be useful to disambiguate phylogenetic signal in saturated inversion histories. Pairwise study of eukaryotic genome arrangement has demonstrated preference for particular types of rearrangement events [65], and methods similar to ours could conceivably be extended to identify selection on arrangement from phylogenies of multi-chromosomal eukaryotic genomes.

A non-phylogenetic, pairwise model of rearrangement by inversion has previously been used to investigate the preference for historic replichore balance in bacteria [66]. Using randomly simulated genome arrangements as a baseline, the authors conclude that historical replichore balance has been significantly maintained in a variety of bacteria, but not all. Our Bayesian method improves on their model by allowing us to gauge more rigorously the degree of statistical confidence and uncertainty in reconstructions of inversion history. Moreover, our method avoids a systematic bias when exploring possible inversion histories. The distribution sampled by the Ajana *et al* method is not uniform over equally parsimonious inversion scenarios, but is skewed to favor particular mutation events. The difference between their sampling distribution and the uniform distribution can grow exponentially in some cases ([67], section 5.2).

## Methods

### Computing Genome Alignments

We used the Progressive Mauve algorithm [68] to compute an alignment of the nine genomes listed in Table 1. Analysis of the resulting alignment indicated that *Y. enterocolitica* 8081 contains substantial gene content differences with respect to the other *Yersinia* genomes, with only 81.5% of an average *Yersinia* genome conserved among all nine taxa. Current Bayesian models of genome arrangement do not model gain and loss of genetic material, thus we removed *Y. enterocolitica* 8081 from further analysis.

An alignment of the eight *Y. pestis* and *Y. pseudotuberculosis* genomes was constructed using the default mauveAligner parameters. The resulting LCBs were inspected using the Mauve alignment viewer and the minimum LCB weight was adjusted to a value which eliminates LCBs consisting of only repetitive elements (LCB Weight 600).

We then computed a full alignment with minimum LCB weight 600, and processed the resulting XMFA alignment file into a permutation matrix in BADGER format (Dataset S1).

### Bayesian Modeling of Genome Rearrangements

We apply the Bayesian model of genome rearrangement by inversion implemented in the BADGER software [69]. BADGER models genomic inversions as a continuous-time Markov process occurring along branches of an unrooted phylogenetic tree which relates organisms. All inversion events are modeled to be equally likely *a priori*. This enables us to calculate the likelihood of a genome rearrangement history mapped onto a tree given the tree and mutation rates, see e.g. [70].

Branch lengths are measured as the number of mutations on a branch, with lengths modeled using an exponential distribution. The mean value of the exponential distribution is given a hyper-prior which creates a strong preference for shorter overall branch lengths and thus assigns higher posterior probabilities to parsimonious inversion histories.

BADGER samples from the joint posterior distribution of tree topologies, inversion histories, and mutation rates using Metropolis-coupled Markov-chain Monte Carlo, also known as MCMC with Parallel Tempering [71]. Accurate inference using MCMC methods requires Markov-chain convergence and adequate mixing. In general, MCMC samplers for genome rearrangement appear to mix very slowly because the likelihood surface can be rugged, and good proposal mechanisms for transitioning between peaks may not exist. The use of heated parallel chains (Metropolis coupling) can alleviate the problem to some extent [72]. The Parallel Tempering method first considers the Bayesian posterior distribution as a Boltzmann distribution at unit temperature. The probability of a particular state *X* in a Boltzmann distribution is defined as
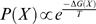
(7)where Δ*G*(*X*) is the free energy, *e* is the natural number and *T* is the temperature. Since we are talking about hypothetical energies and temperatures, we omit the Boltzmann- or gas-constant (*k* or *R*) in the formula. Setting *T* = 1 leads to defining the free energy of a state as
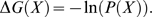
(8)


After defining the free energy for each state, the Parallel Tempering runs several chains with different temperatures, the unheated chain has temperature 1, the heated chains have higher temperatures. All chains converge to their own prescribed Boltzmann distribution, but sometimes they swap states. The swapping is governed by the Metropolis rule ([43]; hence the name, Metropolis-coupled MCMC), which guarantees that swapping does not change the convergence to the prescribed distributions. The probability surface is flat at high temperatures, which provides fast mixing in the state space, while the swappings between the unheated and heated chains allow the possibility that the unheated chain can jump from one local minimum into another one.

In our application to the *Yersinia* LCBs, we used a Metropolis-coupling scheme with temperatures ranging from 1 to 1.18 to ensure adequate mixing. A comparison of runs with 3, 5, 19, and 49 heated chains revealed that only runs with 19 or 49 heated chains discovered all seven parsimonious topologies within 500,000 MCMC steps. Monitoring the log-likelihood plot and comparison among the runs suggests that the chains have converged and mixed sufficiently to support the inferences described in the present work.

To make inference on ancestral genome arrangements, we modified the BADGER C++ code to record inversion histories at each subsample point. Additional software was implemented to summarize the resulting posterior samples of genome arrangement. All software is available from http://bioinformatics.org.au/barphlye.

### Rooting the Tree

Despite exclusion of *Y. enterocolitica* from the genome rearrangement phylogeny, it remains a potentially useful outgroup for rooting the tree using a molecular character such as nucleotide substitutions. Debate rages over the proper method to infer phylogenies using large multi-gene or whole-genome datasets. Recombination, lateral exchange, lineage sorting, and other natural processes can result in a phylogenetic signal that varies widely from gene to gene. One attempt to acknowledge and mitigate the impact of such effects is the recently proposed concordance factor approach, which provides a method to infer the fraction of a genome supporting a given hypothesis of vertical inheritance [73].

We apply Bayesian tree concordance statistics to estimate support for alternative rootings of the phylogenetic network shown in Figure 3. An analysis of 30 randomly selected genes gives an *a posteriori* concordance factor of 19.4 (out of 30, 90% confidence interval [10],[28]) supporting a root on the branch leading to *Y. pseudotuberculosis* IP31758. An alternative rooting on the branch leading to *Y. pseudotuberculosis* IP32768 garners a concordance factor of only 7.5, with a 90% confidence interval of [0,17]. The concordance factor analysis suggests that recombination and lineage sorting in *Yersinia* has caused inconsistent phylogenetic signal throughout the genome, but that a greater fraction of sampled genes support a rooting on *Y. pseudotuberculosis* IP31758. Such frequent large-scale homologous recombination has recently been reported as a common feature in other Enterobacteriacae [74],[75]. Interestingly, the concordance tree splits weakly support placement of *Y. pestis* Pestoides F as a sister taxa to *Y. pestis* KIM, whereas the inversion phylogeny places the Pestoides F lineage as ancestral to the remaining *Y. pestis* with high confidence.

### LCB Lengths and Replichore Balance

Although we discarded *Y. enterocolitica* due to presence of differential gene content, the eight remaining genomes contain some lineage-specific content as well. Differences in gene content imply that observed LCB lengths are different in each modern genome. Moreover, breakpoint regions may contain lineage specific content. To perform inference on ancestral replichore balance with a model that lacks gene gain and loss, it was necessary to assign a length to each LCB and to account for the portion of each chromosome in breakpoint regions. We took a reference-genome approach based on *Y. pestis* KIM, which represents a median in terms of genome size among the eight *Yersinia* genomes studied. We assigned half of each breakpoint region to its neighboring LCB in *Y. pestis* KIM, and took the resulting LCB lengths as representative of all genomes. An average of 6.7% of each modern genome lies in breakpoint regions, and genome size deviates from *Y. pestis* KIM by +/− 3%. Thus, our use of a reference genome introduces some error into estimates of ancestral replichore sizes. In the worst case, the error could be as large as 10%, but the average error is small enough that it does not affect the main conclusions described here.

### Identifying the Origin and Terminus *dif* Site

The origin and terminus of replication in *Y. pestis* KIM was previously identified as occurring at approximately 1 bp and 2.324 Mbp, respectively [25]. Here, the terminus refers to a point on the chromosome where strand-specific oligomer skew shifts abruptly to the opposite strand. Others have reported that the change in oligomer skew typically occurs near the terminus *dif* site [19], and so we use the site of strand bias change as a proxy for the true *dif* site. The *ori* and *dif* sites were assigned in other genomes on the basis of homology to *Y. pestis* KIM. The predicted *dif* site lies in the middle of a large 140 Kbp segment conserved among all *Yersinia* genomes at >95% sequence identity (see Figure 1). Similarly, the predicted origin lies in the middle of a 53 Kbp segment conserved among all *Yersinia* at >95% sequence identity.

Comparison of our origin and terminus predictions to those made by an automated prediction system [76] reveals that our predictions agree with those made by the automated system within 1 kbp in nearly all cases. Discrepancy occurs in the terminus prediction for *Y. pestis* 91001. The discrepancy seemingly results from numerous recent rearrangements having disrupted the signal of strand-specific oligomer skew and in turn confusing the automated system.

### Estimating Significance in Kolmogorov-Smirnov Tests

We report analysis on 30,000 samples from the posterior distribution of inversion histories. We assume that *Yersinia* has one true evolutionary history, and that at most one of the inferred histories represents the true history. As such, when comparing the distributions of quantities of interest, we do so on a per-sample basis using the Kolmogorov-Smirnov test. We take the median *p*-value over the 30,000 tests to be an estimator of the *p*-value which would be obtained had the test been applied to the one true history. We report mean D values as average estimates of the difference between target distributions.

### Permutation Testing for Episodes of Imbalance

We use random permutation to generate a null distribution of the number and duration of episodes of imbalance. A tree sample with inversions mapped onto its branches has one genome arrangement for each leaf (8 in total), one arrangement for each internal node (6 in total), and some number of intermediate arrangements along each branch of the tree. For each sample in the posterior distribution of trees and inversion histories, we assign imbalance values the intermediate genome arrangements in the sample. For each branch of a given tree sample, we generate a permuted distribution by randomly shuffling the imbalance values of intermediate genome arrangements on that branch. We then count the number of transitions to and from imbalance along the original branch and along the branch with permuted values. Thus, the randomly permuted data have the same total number of balanced and imbalanced states with the same balance values, but any clusters of imbalanced states will be uniformly random.

Our permutation approach disregards the actual inversion events, but generates random permutations with the same overall balance values. It is not possible to construct a random permutation of imbalance values by shuffling the inversion events themselves, since overlapping inversion events have strong ordering constraints and violation of these constraints would often change the imbalance values. Moreover, a strategy which samples inversion events uniformly at random would not yield a set of balance values consistent with the set we desire to permute.

### Expected Length of Within- and Inter-Replichore Inversions

**Figure 12:**
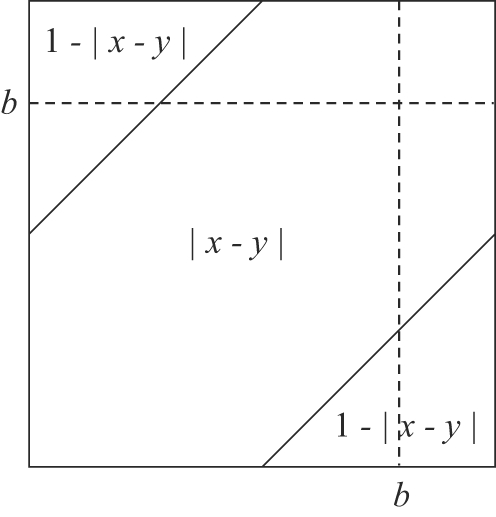
Calculating expected inversion length. The expected length of within- and inter-replichore inversions can be calculated as integral averages of the function min{|*x*−*y*|,1−|*x*−*y*|} over the appropriate areas. Here, 0<*b*<1 is the terminus *dif* site. See the text for more details.

Assume the endpoints of an inversion are in positions *x* and *y*, with *x*, *y*∈[0,1]. The inversion length can be expressed as the function min{|*x*−*y*|,1−|*x*−*y*|}, since the inversion occurs on a circular chromosme of length 1 and for any inversion longer than 0.5, a complementary inversion with shorter length exists. If we assume that the inversion endpoints are uniformly distributed, then the expected length is the integral average of the function min{|*x*−*y*|,1−|*x*−*y*|} over the appropriate area *A*:
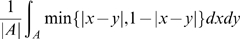
(9)where |*A*| denotes the size of the area. In the case of within-replichore inversions, area *A* is the union of the two squares as delineated by the dashed line of Fig. 12, in case of inter-replichore inversions, *A* is the union of the two rectangles. For simplicity we suppress the full details of integration, and the resulting equations for within- and inter-replichore inversions are given in Equations 4 and 5, respectively.

## Supporting Information

Dataset S1 Genome alignment and genome arrangement data. File - 8way_600from400.badger: A signed gene-order permutation matrix describing the order and orientation of locally collinear blocks (LCBs) as they occur in each of the eight genomes. File - mavvers_8way_600_from_400_perms.600.lcbs: contains the left-end and right-end coordinate of each LCB in the main chromosome of each genome. File - mavvers_8way_600_from_400_aligned.xmfa: contains an XMFA format genome alignment of the eight yersinia that can be viewed in the Mauve viewer. Ensure that the source genbank files (also included in the zip) are located in the same directory to load annotation data. Remaining files: source genome sequence and annotation data.
(39.59 MB ZIP)

Video S1 Evolution of *Y. pestis* KIM. The maximum a posteriori estimate of inversion events on the branch leading to *Y. pestis* KIM. The main circular chromosome is shown as a torus, with the origin and terminus marked. The ancestral left and right replichores are colored blue and green.
(5.28 MB MOV)

Video S2 Evolution of *Y. pestis* Antiqua. The maximum a posteriori estimate of inversion events on the branch leading to *Y. pestis* Antiqua. The main circular chromosome is shown as a torus, with the origin and terminus marked. The ancestral left and right replichores are colored blue and green.
(10.53 MB MOV)

Video S3 Evolution of *Y. pestis* 91001. The maximum a posteriori estimate of inversion events on the branch leading to *Y. pestis* 91001. The main circular chromosome is shown as a torus, with the origin and terminus marked. The ancestral left and right replichores are colored blue and green.
(8.79 MB MOV)

Video S4 Evolution of *Y. pestis* CO92. The maximum a posteriori estimate of inversion events on the branch leading to *Y. pestis* CO92. The main circular chromosome is shown as a torus, with the origin and terminus marked. The ancestral left and right replichores are colored blue and green.
(2.22 MB MOV)

Video S5 Evolution of *Y. pestis* Nepal516. The maximum a posteriori estimate of inversions on the branch leading to *Y. pestis* Nepal516. The main circular chromosome is shown as a torus, with the origin and terminus marked. The ancestral left and right replichores are colored blue and green.
(5.23 MB MOV)

Video S6 Evolution of *Y. pestis* 15–70 Pestoides F. The maximum a posteriori estimate of inversion events on the branch leading to *Y. pestis* 15–70 Pestoides F. The main circular chromosome is shown as a torus, with the origin and terminus marked. The ancestral left and right replichores are colored blue and green.
(7.29 MB MOV)

Video S7 Evolution of *Y. psuedotuberculosis* IP31758. The estimate of inversion events on the branch leading to *Y. psuedotuberculosis* IP31758. The main circular chromosome is shown as a torus, with the origin and terminus marked. The ancestral left and right replichores are colored blue and green.
(3.11 MB MOV)

Video S8 Evolution of *Y. psuedotuberculosis* IP32953. The estimate of inversion events on the branch leading to *Y. psuedotuberculosis* IP32953. The main circular chromosome is shown as a torus, with the origin and terminus marked. The ancestral left and right replichores are colored blue and green.
(1.77 MB MOV)
